# Pain-Induced changes in corticospinal excitability are associated with adaptive changes in muscle coordination

**DOI:** 10.1016/j.ynpai.2026.100212

**Published:** 2026-03-28

**Authors:** Arnaud Duport, Gaspard Diotalevi, Pierre Morel, Flore Le Blanc, Guillaume Léonard, Hervé Devanne

**Affiliations:** a Univ. Littoral Côte d’Opale, Univ. Artois, Univ. Lille, ULR 7369 - URePSSS - Unité de Recherche Pluridisciplinaire Sport Santé Société, F-62100 Calais, France; b Research Centre on Aging, 1036 Rue Belvédère Sud, Sherbrooke, QC J1H 2J7, Canada; c University of Sherbrooke, 3001 12e Avenue Nord, Sherbrooke, QC J1H 5H3, Canada; d University of Sherbrooke, Department of Mechanical Engineering, 2500 Bd de l’Université, Sherbrooke, Canada

**Keywords:** TMS, Input-output curve, Muscle control, EMG, Induced pain, Non-negative matrix factorization

## Abstract

Most pain studies overlook the relationship between corticomotor function changes and coordinated muscle patterns in voluntary movement. A pre-post study was performed to investigate relationships between changes in corticospinal excitability (CSE) and spatial changes in muscle coordination in response to pain. **Methods:** Thirty pain-free participants performed an upper limb pointing task in pain-free then experimental painful conditions. Transcranial magnetic stimulation was used to assess CSE via the deltoid muscle input–output curves (slope, plateau and S_50_). Electromyography was used to record trapezius and deltoid muscle activity. Spatial adaptations of muscle coordination to pain were assessed using non-negative matrix factorization (NNMF), comparing the norm of coordination pattern vectors between pain and no-pain conditions, as well as the cosine of the angle between these vectors. Correlations were examined among pain-induced changes in CSE and muscle coordination. **Results:** Correlations revealed associations between pain-induced S_50_ change and both pain-induced cosine and norm changes (r_S_ = 0.43; *p* = 0.05 for both). Additionally, a correlation was found between pain-induced changes in the coordination pattern’s norm and both the IO curve’s slope (r_S_ = -0.46; *p* = 0.04) and plateau (r_S_ = 0.45; *p* = 0.04). **Discussion:** Overall, this study showed that pain-induced CSE changes were associated with changes in muscle coordination, with participants exhibiting greater CSE changes also showing greater coordination changes.

## Highlights

• Muscle coordination is reorganized in response to experimental pain.
• Pain-induced increase in corticomotor excitability is linked to adaptation of muscle coordination.
• Pain-induced S_50_ changes correlate with muscle coordination pattern norm and angle changes.
• Pain-induced changes in IO slope and plateau correlate with coordination pattern norm change.

## Introduction

Studying the motor system's response to pain is essential for understanding neuromuscular adaptation mechanisms and developing effective therapeutic strategies. These adaptations, although potentially protective in the short term, can contribute to the development of chronic motor dysfunctions. It is already known that the appearance of pain in the musculoskeletal system leads to motor adaptations (Hodges and Tucker 2011) and modifications of motor synergy (Muceli et al., 2014a, Gizzi et al., 2015, Liew et al., 2018). Studies focusing on experimental pain have shown that these adjustments lead to a reduction in the motor response of painful muscle, while modifying the activity of the surrounding muscles, to minimize the impact on function (Madeleine et al. 1999). Other studies have highlighted that these adaptations partly depend on the task (Ervilha et al., 2005, Falla et al., 2007) and involve changes in functional muscle coordination patterns, computed as time-invariant synergies (also called “spatial synergies”) (Cheung et al. 2009). Although most studies investigating motor adaptation use intramuscular injections to induce experimental pain, differences have also already been demonstrated using cutaneous pain induced by capsaicin cream (Dancey et al., 2019, Evans et al., 2021). Furthermore, the effects may differ at the pain site compared to distant locations (Arieh et al. 2022). Muscle synergies, as identified using the non-negative matrix factorization (NNMF) method, represent temporal muscle activation profiles that can be scaled and summed to reconstruct actual muscle activity. Their analysis provides a more comprehensive characterization of a subject’s motor deficits and compensations than kinematics or isolated EMG analysis, offering insights into the flexibility and adaptability of motor patterns (Ivanenko et al., 2005, Clark et al., 2010, Safavynia et al., 2011, Scano et al., 2024). It has been shown that it is more relevant to analyze the joint muscular activity of several muscles of the shoulder girdle rather than the muscles in isolation (Ranaldi et al. 2021). Moreover, pain-induced neuromuscular adjustments may be masked at the kinematic level by compensatory strategies, with similar movements that may be produced by different neuromuscular mechanisms (Safavynia et al. 2011).

At the cortical level, experimental musculoskeletal pain has been shown to modulate the excitability of the primary motor cortex (M1), reflecting adaptive or maladaptive changes in motor control. Several studies using transcranial magnetic stimulation (TMS) have demonstrated that acute pain, whether induced by hypertonic saline injection, thermal stimulation, or pressure, typically leads to a reduction in corticospinal excitability at rest (Le Pera et al., 2001, Farina et al., 2001). However, pain-related changes in M1 are not uniform and can vary depending on the task context, the muscle involved, and the intensity or type of pain (Ingham et al. 2011). This decrease may serve as a protective mechanism to limit movement and prevent further tissue damage, although to our knowledge and following a systematic literature review (PubMed/Scopus/WoS), no study has attempted to relate these changes in excitability to a modification of muscle synergies or coordination in the face of pain. Yet we know that changes in muscle coordination during upper limb movements have been associated with modulations in excitability during the motor preparation phase, suggesting a role for the primary motor cortex (M1) in shaping these adaptations (Augenstein et al. 2024). This link is further supported by evidence that CSE increases during tasks demanding greater precision and fine muscle coordination compared to analytic isolated task (Kouchtir-Devanne et al. 2012). Demonstrating that pain-induced changes in corticospinal excitability are linked to alterations in muscle coordination would establish a direct connection between cortical adaptations and motor reorganization. This could enhance our understanding of how pain disrupts the motor cortex and the impact on the motor adaptation.

The aim of this study was to use an NNMF coordination-based approach to investigate possible interrelationships between pain-related changes in muscle coordination and corticospinal excitability during a pointing task. We hypothesize that pain adaptation of muscle coordination will be linked with changes in M1 excitability.

## Methods

### Ethics and approval

This pre-post study was approved by the ethics committee *Ile de France VI* (Reference 22.00817.000057) on April 8, 2022 and conducted according to the principles of the Declaration of Helsinki (World Medical Association 2013). Informed consent was obtained from all individual participants included in the study. The authors confirm that all methods were performed in accordance with the relevant guidelines and regulations. The trial was registered on ClinicalTrials.gov (NCT05396820).

### Participants

Thirty healthy subjects (16 male and 14 female) voluntarily participated in this study after providing their written and informed consent. Participants were recruited by posting notices around the research center and by word of mouth. Testing took place from September 5th, 2022, to October 28th, 2022, in Eurasport facilities (Loos, France).

Participants had to meet the following criteria: be over 18 years old, demonstrating proficiency in French, no cognitive impairments, abstain from recreational substances, tobacco, and short-acting analgesics for at least 6 h prior to data collection.

Exclusion criteria were as follows: history of chronic pain and/or motor or sensory disorders, contraindications to transcranial magnetic stimulation (e.g., presence of implanted medical devices), shoulder pathology, pregnancy, allergies to capsaicin.

### Course of the study

**Fig. 1:**
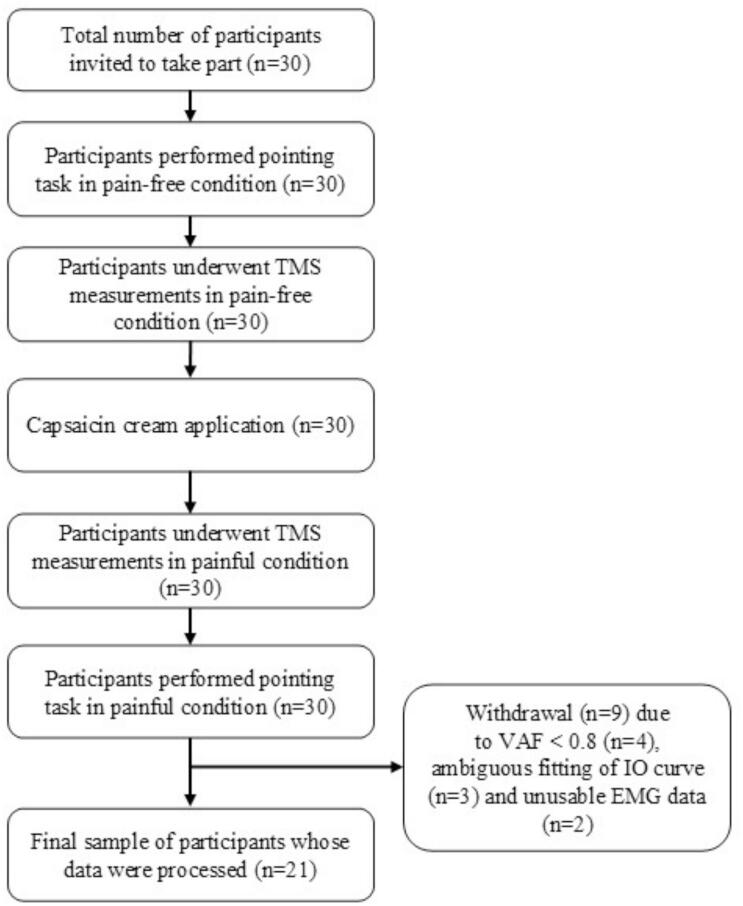
Flowchart of the study.

Participants underwent a single experimental (∼2 h) session, which took place as follows: first, they were asked to perform a pointing task, followed by an evaluation of CSE. Then, experimental pain was then induced by application of capsaicin on the deltopectoral groove. Finally, a second assessment of CSE was done, followed by a pointing task, while participants were in painful condition (Fig. 1). To focus on the core motor coordination underlying the pointing task, only the anterior deltoid and upper trapezius were analyzed. These muscles respectively drive shoulder flexion (Elzanie and Varacallo 2025) and scapular stabilization (Paine and Voight 2013), capturing the essential coordination of arm elevation and reach. The measurements were carried out by experienced researchers in TMS and EMG.

### Pointing task and EMG recording

Participants were instructed to sit on a chair with their right forearm resting on a table in front of them and asked to keep their feet flat on the ground while not resting their back on the backrest. The table and chair were maintained in the same position for all participants. A visual cue, corresponding to the initial index position, was drawn on the midline of the table. The pointing target was placed at the top of a 10 cm stick on the midline, at a distance corresponding to about 80% of the subject’s maximum arm extension, without allowing trunk movement. Surface electrodes (Trigno Wireless, Delsys, USA) were placed on clean, dry skin in the middle of the belly of the AD and UT muscles, taking into account the direction of the muscle fibers. Correct electrode positioning was assessed by checking the EMG signals while the subject performed either arm flexion (AD) or shoulder elevation (UT). The experimental task consisted of the execution of 27 voluntary movements in a spontaneous manner, prompted by verbal instructions from the experimenter every 5–7 s to allow for complete muscle relaxation between repetitions, without any emphasis on promptness or speed, and moving the trunk as little as possible. Each participant was given time to familiarize themselves with the task prior to the experimental trial (few trials). After completing each movement and returning to the initial position, the experimenter checked that the recorded muscles were in a relaxed state and that the instructions for repositioning the finger and elbow had been followed.

Surface EMG signals were amplified (×1,000) and filtered in the 10–1,000 Hz bandwidths before digital sampling at 2 kHz with a 1401 Micro MKII device (Cambridge Electronic Design, UK). Electromyographic signals from the AD and UT muscles were pre-processed using a customized MATLAB algorithm (Version R2018a, USA). The linear trend was removed before filtering each signal with a fourth order zero-lag band-pass Butterworth filter set between 20 Hz and 400 Hz to remove motion artifacts (low frequencies) and noise (high frequencies) from data. Full wave rectification and smoothing through a fourth order zero-lag low-pass Butterworth filter (cut-off frequency 5 Hz) then yielded EMG envelopes. Movement onset was determined from EMG data as the last local minimum in EMG envelope amplitude preceding observable movement from kinematic data. We kept the onset time of the first of the two muscles to contract as the global movement onset time. If no local minimum was to be found, the algorithm defaulted to the mean activation time difference from kinematic data for the given participant and condition across repetitions. Signals were then cut from the onset of movement to the index reaching the target for each repeated trial and resampled to 2,000 evenly spaced time points to standardize the temporal dimension across trials and muscles, facilitating NNMF computation and simplifying calculations. Data matrices were then horizontally concatenated for each participant among both pain conditions rather than averaged to increase reconstruction quality (Liew et al. 2018). Finally, EMG values were normalized over concatenated trials in both conditions.

The normalization method does not significantly affect extracted spatial synergies, but alters the variance accounted for (VAF), affecting the number of extracted synergies (Kieliba et al. 2018). However, this was not a factor in this study as only one synergy, therefore considered as a coordination pattern, can be extracted from our dataset. The most common method in current literature is the normalization of each muscle amplitude by its maximum value across concatenated trials (Zhao et al. 2023); the second, third and fourth most common methods are normalization by unit variance, median value or average maximum among individual trials, respectively (Ortega-Auriol et al. 2025). We performed each of these normalization methods across concatenated trials. We also tried normalizing concatenated EMG signals by their mean and kept the method best suited for our needs, based on our VAF criteria detailed in “Outcomes/Muscle coordination” section.

### TMS measurements

CSE of the AD muscle was assessed during a standardized isometric shoulder flexion at 10% (± 2%) of the participant’s maximal voluntary activity using visual feedback, to facilitate reliable MEP recordings. This weak contraction was consistently maintained in both pre- and post-pain induction conditions to ensure comparability between them. Furthermore, pain-induced CSE modulation follows the same trend in both active and passive settings. (Lewis and Byblow, 2002, Chye et al., 2010, Roosink and Zijdewind, 2010). A 1401 + device (CED, UK), coupled with customized Signal® software (CED, UK), was utilized to display the root mean square (RMS) level of the low-passed (100 Hz) AD EMG. Input output (IO) curves of AD motor evoked potentials (MEPs) versus TMS intensity were measured to assess changes in CSE. Participants sat comfortably in an armchair with their feet on a footrest and their head on a headrest. The coil, oriented tangentially to the skull, was placed over left M1 with the handle at an angle of 45° in the medial sagittal plane to induce a posterior-anterior electric current using monophasic pulse. The optimal hotspot for the right AD was determined by moving the coil around C3 (10–20 system), cm by cm, until the largest MEP was recorded. The coil was then maintained on the hotspot using a mechanical articulated arm. To construct the IO curve, at least 8 MEPs were recorded at each stimulus intensity, ranging from subthreshold intensity (3% below threshold) to the intensity evoking the largest response or reaching maximum stimulator output (MSO). Acquisitions were done incrementally, with steps of 3 to 5% of the MSO.

### Experimental pain

We applied a dose of 0.06 ml of a topical 1% capsaicin cream (prepared on request by Gentès and Bolduc, © Familiprix pharmacists, with 1% Capsaicin Powder USP and 99% Dermabase Cream) on an area of approximately 4 × 4 cm on the participant’s right delto-pectoral groove on intact and non-irritated skin, as is frequently done in research (Martel et al., 2017, Segning et al., 2022). A pain evaluation was made every 1–2 min until it was stabilized. Pain stabilization (assessed when participants rated the same intensity of pain on 3 consecutive measurements) occurred approximately 10 min after capsaicin application, reaching a mean intensity of 6.2 ± 1.7/10 (mean ± SD) on the verbal numerical pain scale ranging from 0 = “no pain” to 10 = “the worst pain imaginable”.

## Outcomes

### Muscle coordination

Only one coordination pattern could be extracted from our dataset on two agonist muscles, as two would perfectly recreate the original dataset without reducing its dimension thus defeating the purpose of extracting synergies.

**Fig. 2:**
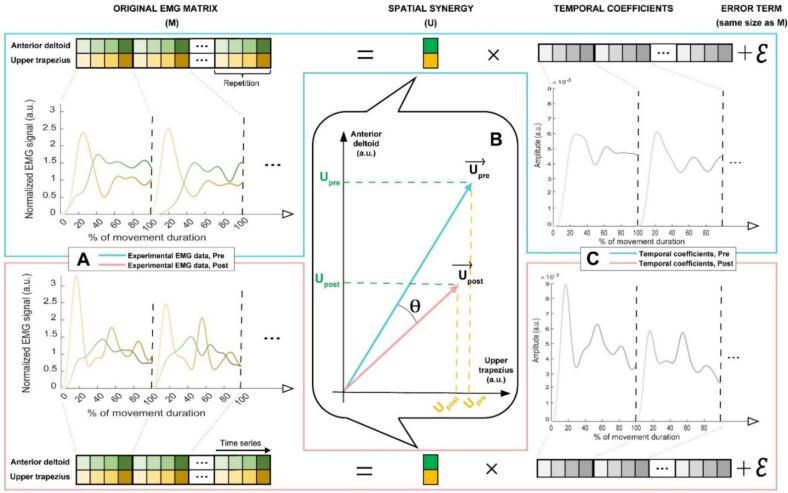
Illustrative decomposition of concatenated EMG signals (M) into spatial coordination (U), temporal coefficients (C) and an error term (ε) by application of the non-negative matrix factorization (NNMF) method. Only three concatenated trial repetitions of four measures are shown for ease of representation in matrix M. The full EMG dataset can be represented as EMG curves (A) for each muscle (anterior deltoid in green and upper trapezius in orange yellow) in the pain-free (framed in blue) or painful (framed in pink) condition. Repetitions are separated by black dashed lines. Spatial coordination pattern (U) vectors (B) quantify the intensity and distribution of muscle activation, revealing the angle of change (θ) between the coordination pattern' vectors in the two conditions. The time coefficients for each condition form a common curve shared by both muscles (C). (For interpretation of the references to colour in this figure legend, the reader is referred to the web version of this article.)

The model includes m = 2 muscles (trapezius and deltoid), s = 1 coordination pattern, k = 1 task (pointing task) of t = 2000 samples repeated r = 27 times for every participant. The spatial coordination pattern (***U***, **m** rows, **s** columns) and temporal coefficients (***C***, s columns, **k** × **t** × **r**) were identified from pre-processed EMG signals (***M***, m rows, **k** × **t** × **r** columns) (Eq. (1) by using the base non-negative matrix factorization method (NNMF) (Chiovetto et al. 2013) built in MATLAB which uses the alternating least squares algorithm. This optimization algorithm was run 20 times to ensure the lowest reconstruction error (ε,mrows,k×t×rcolumns) possible (Eq. (1). Fig. 2 illustrates the decomposition of an NNMF.

(1) M=UC+ε

The reconstructed EMG matrix (Eq. (2) is usually used to compute the goodness of fit as the VAF (Eq. (3). Note that, contrary to a determination coefficient, the total sum of squares is not centered. VAF was computed both globally and for each individual muscle (Roh et al. 2013) (“muscle” equals Eq. (1) or Eq. (2) instead of a sum in Eq. (3) in that case) for each normalization method. As the number of coordination pattern cannot be optimized to obtain satisfactory VAF values for all participants (analysis capped at 1 coordination pattern and 2 muscles), the number of participants meeting a simple threshold of 0.8 for each 6 VAF values (individual VAF for both muscles as well as global VAF in pre and post conditions) was recorded for each normalization method. The method that had the highest number of participants meeting our VAF criteria was kept and participants that the NNMF could not adequately model were excluded from further analysis.

(2) $$\hat{M}=U∙C$$

(3) $$\mathrm{VAF}=1-\frac{\sum_{\mathrm{muscle}=1}^{2}\sum_{\mathrm{time}=1}^{t}{\left(M_{\mathrm{muscle},time}-{\hat{M}}_{\mathrm{muscle},time}\right)}^{2}}{\sum_{\mathrm{muscle}=1}^{2}\sum_{\mathrm{time}=1}^{t}{M_{\mathrm{muscle},time}}^{2}}$$

Muscle coordination pattern parameters compared between pain-free and induced pain conditions were thus the cosine of the angle between the spatial coordination patterns ***U*** in the two conditions and their Euclidian norm’s difference between conditions. A decrease in the cosine of the angle θ from 1 reflects a change in the coordination pattern, indicating a modification in the relative contributions of the muscles involved. In contrast, differences in the norms of the coordination pattern vectors approximately reflect variations in the overall EMG amplitude, thereby indicating changes in the intensity of muscle activation between pain conditions.

### Corticospinal excitability

The excitability of M1 was assessed using the slope and the S_50_ of the IO curve. The average peak-to-peak MEP amplitude was plotted against stimulus intensity, and the data points were fitted to a Boltzmann sigmoidal equation using GraphPad (version 9.0.0, USA) (Devanne et al. 1997). The equation (Eq. (4) describes the relationship between MEP amplitude and stimulus intensity (S):

(4) $$MEP\left(S\right)=y_{0}+\frac{\mathrm{ME}P_{\mathrm{MAX}}}{1+e^{\frac{S_{50}-S}{k}}}$$

The equation (4) comprises four parameters: MEP_MAX_ is the plateau of the curve; S_50_ represents the stimulus intensity needed to elicit 50% response of the maximum; k, and its reciprocal (the slope, 1/k, reflecting the neural recruitment rate, assessing corticospinal excitability dynamics), is proportional to the maximum slope of the curve, occurring at S_50_; y_0_ corresponds to the baseline of the curve. We employed the maximum slope value at S_50_ as an indicator of the steepness of the curve. Slope and the S_50_ have been chosen because they represent a global index of M1 excitability (Stagg et al. 2011).

### Statistical analysis

GraphPad was used for statistical analyses. First, a linear mixed-effects model was used to assess the effect of repeated trials on EMG (peak amplitude and area) in the pain-free condition. Trial index was included as a fixed effect to evaluate potential learning or adaptation across repetitions, while subject was included as a random intercept to account for inter-individual variability. For what follows, because the data were not normally distributed (as revealed by Shapiro-Wilk tests), non-parametric correlation tests were used. Spearman correlation was performed between pre-post difference in muscle coordination pattern (norms and cosine) and curve’s parameters (slope, plateau and S_50_) to investigate whether changes in muscle coordination pattern were associated with corticospinal excitability properties. To enhance replicability, reduce false positives, and uphold scientific rigor, the threshold for statistical significance is lowered to *p* < 0.005 and *p*-values ranging between 0.05 and 0.005 are considered “suggestive” (Benjamin et al. 2018).

Post-hoc power analyses were conducted for each Spearman correlation using Fisher's r-to-z transformation (Jerrold-H-Zar, 1984, Schober et al., 2018). The observed Spearman coefficient (r_S_) was first converted to a z-score via z = arctanh(r_S_), with an associated standard error of SE = 1/√(N − 3). Achieved power was then computed as the probability of rejecting the null hypothesis given the observed effect, using the non-centrality parameter λ = arctanh(r_S_) × √(N − 3) and assuming a two-tailed test at α = 0.05 (Eq. (5) where Φ denotes the standard normal cumulative distribution function. The minimum sample size required to achieve 80% power was estimated by solving equation (Eq. (6), where z_α_/2 = 1.96 (two-tailed, α = 0.05) and z_β_ = 0.84 (1 − β = 0.80) (Cohen, 1988, Bonett and Wright, 2000).

(5) $$1-\beta =1-\Phi \left(z_{\frac{\alpha }{2}}-\lambda \right)+\Phi \left(-z_{\frac{\alpha }{2}}-\lambda \right)$$

(6) $$N={\left[\frac{z_{\frac{\alpha }{2}}+z_{\beta }}{\mathrm{arctanh}r_{S}}\right]}^{2}+3$$

Exploratory Spearman rank correlations were computed between pain-induced changes in TMS IO parameters and motor coordination metrics (m = 6 tests). Benjamini-Hochberg FDR correction (q = 0.05) was applied across all tests for multiplicity control (Benjamini and Hochberg 1995). Uncorrected p-values are reported alongside FDR-adjusted values as this is a hypothesis-generating analysis without prior effect sizes (Rothman, 1990, Althouse, 2016). These findings need to be confirmed by other studies with sufficient statistical power.

## Results

### Participants’ characteristics

**Fig. 3:**
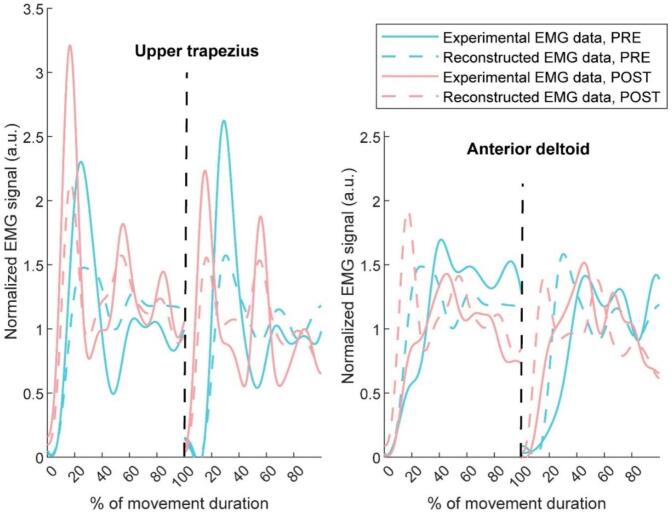
Example of original (solid lines) and reconstructed EMG traces (dashed lines) of the upper trapezius (left) and anterior deltoid (right) in pain-free (PRE, in blue) and painful condition (POST in pink) for two concatenated trials separated by black dash lines. (For interpretation of the references to colour in this figure legend, the reader is referred to the web version of this article.)

VAF was used to select the best normalization method for data. The mean value normalization method was found to let the NNMF model fit the largest proportion of participants with a VAF threshold of 0.8 (Table 1). Fig. 3 shows an example of comparison of reconstructed EMGs with original EMGs, for both muscles.

**Table 1:** Variance accounted for (VAF) depends on normalization methods.

**Normalization method**	**Max value**	**Median value**	**Average Max Value**	**Unit Variance**	**Mean value**
VAF (mean ± SD)					
AD pre	96.7 (2.5)	80.4 (14.5)	95,1 (1,7)	94,1 (2,9)	88,2 (8,4)
UT pre	79.7 (13.8)	96.2 (1.3)	83,9 (11,5)	84,6 (12,5)	92,2 (3,0)
Global pre	92,8 (3,6)	92,3 (3,1)	91,3 (3,8)	91,5 (3,9)	90,6 (4,6)
AD post	96.0 (3.4)	83.7 (15.1)	94,9 (2,1)	92,9 (4,1)	89,4 (8,5)
UT post	82.7 (13.5)	95.7 (2.0)	85,5 (11,7)	87,0 (13,0)	92,2 (3,3)
Global post	92,7 (4,1)	92,4 (3,2)	91,7 (4,3)	92,0 (4,4)	91,1 (5,0)
Number (and %) of participants meeting all VAF criteria	14 (46.7%)	16 (53.3%)	18 (60.0%)	17 (56.7%)	26 (86.7%)

VAF: variance accounted for; AD: Anterior Deltoid; UT: Upper Trapezius; pre: pre pain induction; post: post pain-induction.

Using the mean value normalization method, nine participants were withdrawn from the analysis: four due to a low VAF (< 0.8), three due to ambiguous fitting between TMS measurements and IO curves, and two due to unusable EMG data (motion artifacts and signal loss). There was no significant learning effect on pain-free trials (both *p* > 0.49). Among the 21 participants (9 females, 12 males; mean age = 28.10 ± 5.09 years; all right-handed), pain resulted in an average decrease in S_50_ of 0.43% MSO (95% CI, −2.787 to 1.927), an average increase in slopes of 0.01 mV/% MSO (95% CI, −0.054 to 0.074) and an average decrease in plateau of 0.09 mV (95% CI, −0.379 to 0.210). Additionally, pain resulted in an average decrease of the norm of vectors of 2.16 (arbitrary units, 95% CI, −4.23 to −0.08), and there was an average cosine of 999.23 × 10^−3^ (arbitrary units, 95% CI, 998.7 × 10^−3^ to 999.8 × 10^−3^) (Table 2).

**Table 2:** Muscle coordination pattern data and corticospinal excitability adaptation to pain.

**Variables**	**Mean (SD)**
**Muscle coordination pattern**	
*Cosine of the angle between vectors before and during pain (10^-3^a.u.)*	999.23 (1.23)
*Difference of norm before and during pain (a.u.)*	−2.16 (4.56)
**Corticospinal excitability**	
*Difference of S_50_ before and during pain (%MSO)*	−0.43 (5.51)
*Difference of slopes before and during pain (mV/%MSO)*	0.01 (0.15)
*Difference of plateau (mV)*	−0,09 (0,63)

SD: Standard deviation, MSO: Maximum Stimulator Output, mV: millivolt.

### Correlation between pain-induced changes of corticospinal excitability and muscle coordination

**Fig. 4:**
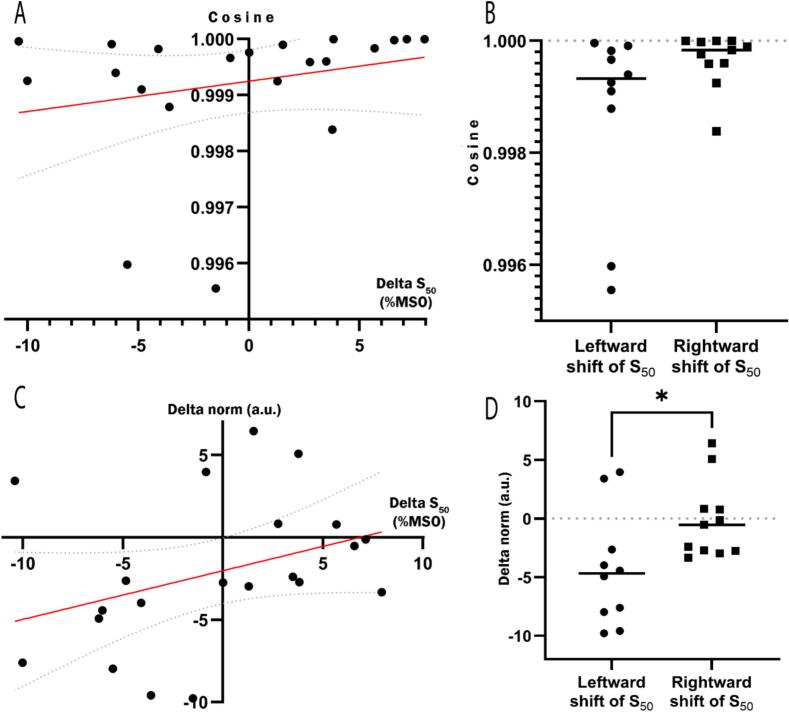
Spearman correlation between pain-induced S_50_ change and between-conditions for cosine (A) and norm (C) (rs = 0.43 [95% CI, −0.02 to 0.73]; p = 0.05 for both). The linear regression line is in red and the 95% confidence bands around the regression line is curved gray dotted line; Mann-Whitney tests comparing left and rightward shift of S_50_ for cosine (p = 0.07 [95% CI, −0.00 to 0.00]) (C), and norm (p = 0.02 [95% CI, 1.12 to 8.39]) (D). (For interpretation of the references to colour in this figure legend, the reader is referred to the web version of this article.)

Spearman correlations suggested an association between pain-induced change on S_50_ and both the difference in norm of coordination pattern vectors and the cosine between these vectors (r_S_ = 0.43 [95% CI, −0.02 to 0.73]; *p* = 0.05 for both), indicating that a leftward shift of S_50_ toward low %MSO (increased CSE) was associated with more changes in muscle coordination (Fig. 4A and 4C, respectively). To illustrate in an exploratory way the impact of a S_50_ shift direction on cosine and norm, we performed two post-hoc non-parametric Mann-Whitney tests comparing leftward and rightward shifts. These analyses were not pre-planned and should be interpreted with caution. A leftward shift of S_50_ tended to have more impact on cosine than a rightward shift, though this did not reach the level of significance (*p* = 0.07 [95% CI, −0.00 to 0.00]), while a leftward shift of S_50_ had a significant reducing effect on norm than a rightward shift (*p* = 0.02 [95% CI, 1.12 to 8.39]) (Fig. 4B and 4D, respectively).

**Fig. 5:**
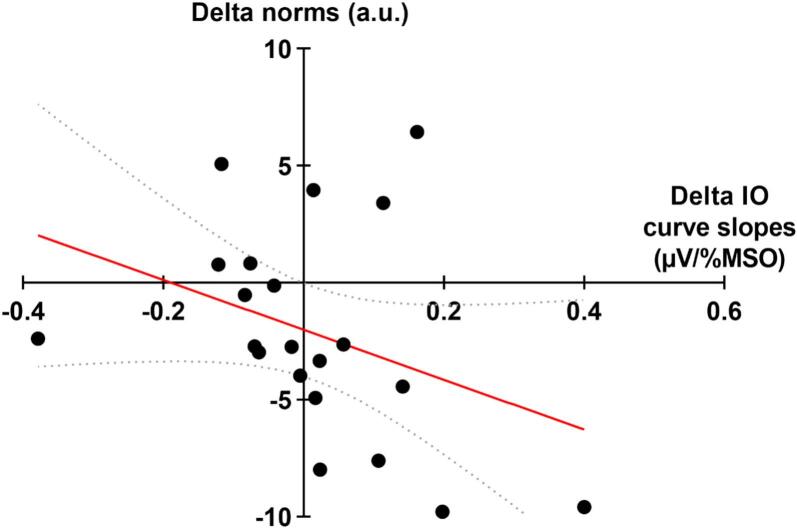
Spearman correlation between pain-induced change of IO curves’ slopes and coordination pattern’s norm (r_S_ = -0.46; p = 0.04). The linear regression line is in red and the 95% confidence bands around the regression line are curved gray dotted line. (For interpretation of the references to colour in this figure legend, the reader is referred to the web version of this article.)

**Fig. 6:**
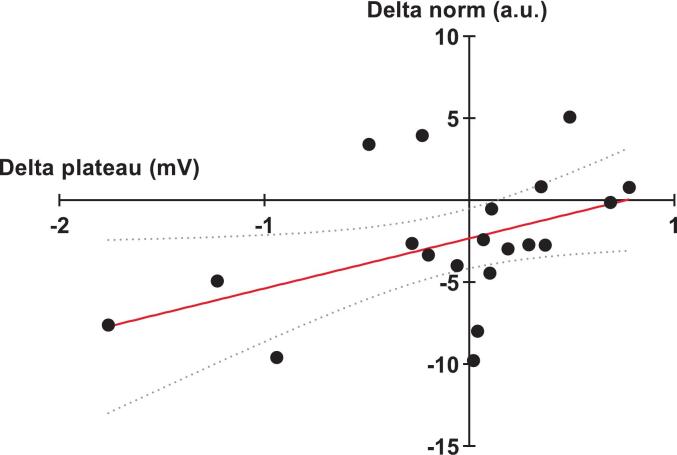
Spearman correlation between pain-induced change of slopes’ plateau and coordination pattern’s norm (r_S_ = 0.45; *p* = 0.04). The linear regression line is in red and the 95% confidence bands around the regression line are curved gray dotted line. (For interpretation of the references to colour in this figure legend, the reader is referred to the web version of this article.)

Spearman correlations also suggested an association between pain-induced changes in coordination pattern’s norm and both changes in plateau (r_S_ = 0.45 [95% CI, 0,02 to 0,75]; *p* = 0.04) and changes in IO curve’s slope (r_S_ = -0.46 [95% CI, −0.75 to −0.02]; *p* = 0.04), suggesting that the plateau and the coordination pattern’s activation vary jointly (Fig. 5) while an increase recruitment rate was associated with a decrease of the coordination pattern’s activation (Fig. 6). No correlations were found between pain-induced change of IO curve’s slope and cosine (r_S_ = -0.28 [95% CI, −0.61 to 0.22]; *p* = 0.23) nor between pain-induced change plateau and cosine (r_S_ = 0.17 [95% CI, −0.30 to 0.58]; *p* = 0.46).

Post-hoc power analyses were conducted for each Spearman correlation using Fisher's r-to-z transformation (N = 21, α = 0.05, two-tailed); results are summarized in Table 3.

**Table 3:** Post-hoc power analysis and minimum sample size required.

**Correlation**	***r*_S_**	***p* value**	**Sig. uncorrected**	***p*-FDR**	**Sig. FDR**	**Power**	**N required**
ΔS_50_ × Δnorm coordination pattern	0.43	0.05	Yes	0.07	No	50%	41
ΔS_50_ × cosine	0.43	0.05	Yes	0.07	No	50%	41
Δnorm coordination pattern × Δplateau	0.45	0.04	Yes	0.07	No	54%	37
Δnorm coordination pattern × ΔIO slope	−0.46	0.04	Yes	0.07	No	56%	35
ΔIO slope × cosine	−0.28	0.23	No	0.28	No	23%	98
Δplateau × cosine	0.17	0.46	No	0.46	No	11%	270

FDR: false discovery rate; r_S_: Spearman correlation coefficient; Δ: pain-induced change. N required = minimum sample size to achieve 80% power (1 − β = 0.80, α = 0.05, two-tailed). Note: No correlations survived FDR correction (all *p*-FDR > 0.05). Suggestive uncorrected trends (r_S_ = ±0.43–0.46, p < 0.05) between coordination pattern normalization and IO curve parameters (plateau/slope) warrant confirmation in future powered studies.

## Discussion

To our knowledge, this is the first study to link changes in corticospinal excitability and changes in motor behaviour as reflected by muscle coordination, in context of pain. Pain-induced S_50_ changes correlated with both cosine and norm differences in muscle coordination, suggesting that increased CSE is linked to muscle coordination alterations. Additionally, pain-induced changes in coordination pattern norm correlated with both IO slope and plateau changes, further supporting a CSE– muscle coordination relationship.

Induced pain had a heterogenous impact on CSE, with increases and decreases depending on the parameters observed. By separating the sample into two groups for each parameter, it is possible to see that certain subjects show an increase in CSE while others show a decrease. A global increase in CSE (e.g. leftward shift S_50_ or increase slope), associated with a modification of the muscle coordination, while decrease in CSE (e.g. rightward shift S_50_ or decrease slope), is rather associated with no change in muscle coordination. Most of the time, induced pain lead to a decrease CSE despite the evidence are limited (Rohel et al. 2021). In our study, people showing a decrease in CSE were also those who maintained their muscle coordination as before pain induction. Conversely, people showing an increase in CSE were also those who modified their muscle coordination, with changes of the relative contributions of the muscles involved in the pointing task, as well as the EMG pattern activation intensity. This variability in our sample is consistent with previous data (Liew et al. 2018), and could be explained by the fact that some individuals require prolonged exposure to pain to implement new adaptive coordination or that changes are more gradual, and therefore less visible in this type of pre-post design. Other studies shown that psychological factors could drive motor adaptations (Hodges and Moseley 2003). Furthermore, variability in CSE responses to pain has been documented in healthy participants (Martel et al. 2017) and has been linked to fear-avoidance behavior, including kinesiophobia (Summers et al., 2020, Duport et al., 2024, Duport et al., 2022). In these latter studies, individuals with the lowest levels of kinesiophobia exhibited the greatest CSE inhibition, whereas those with the highest levels of kinesiophobia showed reduced inhibition or even increased CSE, which might be related to processes supporting motor skill adaptation (Tallent et al. 2021). This association between high kinesiophobia and a lack of inhibition was potentially linked to the increase of CSE observed during the body-focused attention (Matsumoto et al. 2024) underlying the concept of fear-related pain avoidance. Given that kinesiophobia has been identified as a potential predictor of chronic pain and disability (Leeuw et al. 2007), it could be hypothesized that the increase of CSE in response to pain, combined with the sudden changes in muscle coordination observed in some of our participants, may contribute to maladaptive motor adaptation mediated by kinesiophobia. Considering the findings of our study, we speculate that an increase in CSE in response to pain, reflected by leftward shift of S_50_, as well as increase of slope and plateau, and associated with the greater changes in muscle coordination observed immediately after pain induction, could represent a maladaptive motor response and might contribute to the development of chronic pain and disability. However, given the cross-sectional and acute nature of our design, this interpretation remains hypothetical and would require prospective investigation. A sudden change of muscle coordination in response to pain may lead to several adverse outcomes. Compensatory mechanisms may arise, increasing the risk of secondary injuries by placing excessive stress on other muscles or joints (Hodges and Tucker, 2011, van Dieën et al., 2017). Such rapid modifications may render movements less efficient, leading to increased fatigue and diminished performance (Steele et al., 2015, Kakehata et al., 2024). Altering muscle coordination in response to pain may impede the body's intrinsic healing mechanisms (Boudreau et al. 2010). These changes may involve redistribution of activity within and between muscles, modifying mechanical behavior to protect tissues from further harm (van Dieën et al., 2017, Manickaraj et al., 2017), but may also potentially leading to long-term consequences such as increased load and decreased movement variability (Hodges 2011).

Although a change occurs at the cortical level, it is also possible that a part of pattern changes was driven at the segmental level of the spinal cord. Several studies have shown that motor synergies involved in a task such as ours (reach-to-grasp movements) can be conceived as weighted combinations of only a few main muscle activation patterns (Mason et al., 2001, d’Avella et al., 2006). These patterns of activation are function-specific, and show overlaps within M1 (Massé‐Alarie et al. 2017). When faced with acute pain, the central nervous system may be prompted to adopt an alternative pattern of muscle activation driven by cognitive influences (Liew et al. 2018), with fine local adjustments in response to peripheral inputs. Nevertheless, one study reported that induced pain-related descending inhibitory control differentially modulates withdrawal reflex synergies across muscles: distal muscles exhibited reduced activation within a narrow time window (118–156 ms), whereas proximal muscles showed a more sustained reduction at longer delays (150–250 ms), suggesting a greater supraspinal contribution to the control of proximal muscles (Jure et al. 2019), such as AD and UT. However, most acute pain studies (Muceli et al., 2014a, van den Hoorn et al., 2015, Gizzi et al., 2015) document synergy reorganization without directly measuring spinal mechanisms, so direct evidence for spinal involvement is lacking.

### Limitations

The present study was limited by a small sample size (N = 21). Post-hoc power analyses using Fisher's r-to-z transformation revealed that achieved power ranged from 50% to 56% for significant correlations (r_S_ = 0.43–0.46), and from 11% to 23% for non-significant ones (r_S_ = 0.17–0.28), remaining well below the conventional 80% threshold. Although Benjamini-Hochberg correction was applied (*p*-FDR = 0.075–0.46), uncorrected trends (p < 0.05) are reported as hypothesis-generating. Adequately powered replication would require samples of 35–41 participants for the observed significant effects, and up to 98–270 for the smaller non-significant ones. Additionally, although sex differences in pain processing and corticomotor excitability are documented in the literature, no sex-stratified analyses were conducted, as the sample size would render such comparisons underpowered and uninterpretable. Our findings should therefore be considered as preliminary, and the null results cannot be interpreted as evidence of absence of association.

As more than two muscles are involved in the pointing task, analyzing the coordination patterns of a larger muscle array would be necessary to ascertain the relationship between muscle coordination and CSE. Furthermore, adding several muscles to the NNMF model would provide greater flexibility in the number of extractable components and allow for a better-fitting model while avoiding overestimation of the VAF (Damiano 2015). However, only datasets for the trapezius and anterior deltoid muscles were available for this exploratory study. Importantly, with only two muscles and a single extracted component, the NNMF decomposition constitutes a degenerate case in which the synergy vector is reduced to a two-dimensional weighted combination with no genuine redundancy reduction, a limitation that restricts the present findings to a two-muscle coordination pattern rather than a true multi-muscle synergy. Generalization to the broader construct of muscle synergy reorganization therefore requires validation in larger muscle arrays (Burkholder and van Antwerp 2013).

A further limitation concerns the fixed order of conditions, with the pain-free baseline always preceding the pain condition. Potential confounds including fatigue, task learning, attentional drift, and habituation to the experimental setting cannot be fully excluded. This fixed order was however an unavoidable consequence of the pharmacological properties of topical capsaicin, whose pain-inducing effect persists for several hours after application, precluding condition reversal within a single session. Conducting two separate counterbalanced sessions would have introduced a different source of variability, namely inter-session differences in baseline corticospinal excitability, muscle coordination, and pain sensitivity, which would have been equally difficult to control. Furthermore, as the primary analyses were based on correlations between pain-induced changes rather than direct pre-post group comparisons, uniform order effects are unlikely to have distorted the inter-individual covariance structure underlying the reported associations. Differential order effects across participants nonetheless remain a residual confound, and future studies should incorporate designs that better control for temporal confounds while maintaining ecological validity.

Also, the present experimental pain model involved cutaneous nociceptor activation via topical capsaicin (Paintal, 1960, Ro and Capra, 1999), while several previous studies on corticomotor excitability changes during experimental pain used intramuscular hypertonic saline, recruiting deep group III and IV afferents (Chang et al. 2026). These afferent populations differ in their spinal and supraspinal projections, leading to potentially distinct modulatory effects on motor cortical output. Nevertheless, both pain modalities converge at multiple central levels, such as the spinal cord (Mørch et al., 2007, Li and Baccei, 2017), thalamus (Dong et al. 1978) or motor cortex (Mercier and Léonard 2011), and both have been shown to transiently inhibit corticomotor excitability (Le Pera et al., 2001, Farina et al., 2001, Cheong et al., 2003, Fierro et al., 2010, Sacco et al., 2014, Schabrun et al., 2015, Larsen et al., 2018, Dancey et al., 2018, Nishi et al., 2024). Therefore, caution should be taken when extrapolating to deep musculoskeletal or chronic pain conditions.

As the motor task chosen in this study is not cyclical in nature, the datasets are discontinuous, but the reconstructed signals still achieve satisfactory VAF values. Further research would be required to make sure that NNMF processing of discontinuous EMG datasets does not cause unexpected adverse effects. Normalizing data to the maximum value of EMG signals across all trials of the same pain condition is the most common approach (Zhao et al. 2023). Mid-analysis checks showed this method could skew data, with some participants exceeding the 0.9 global VAF threshold while one muscle’s VAF fell below 0.1. Using the mean of all maximum amplitudes across concatenated trials proved more robust, especially with more trials. The unit variance method was unsuitable for our dataset, though it reportedly reduces bias toward high-variance EMG signals (Roh et al. 2013). Surprisingly, normalizing signals by their mean value proved much more reliable than using their median, although the latter is less easily skewed by a low proportion of high values.

Results confirm that the normalization method affects VAF (Kieliba et al. 2018), indicating that with more instrumented muscles, it could influence the number and interpretation of extracted synergies and muscle coordination patterns. While normalization facilitates inter-study EMG comparisons, it may impact synergies and muscle coordination patterns extraction and should be applied with caution.

## Conclusions

This study provides novel evidence that pain-induced changes in corticospinal excitability are associated with adaptations in muscle coordinations. Specifically, increased excitability, as reflected by leftward shifts of S_50_, steepening of the slope and increased plateau of the input–output curves, correlated with alterations in the direction and amplitude of coordination pattern vectors. These findings highlight a functional link between corticomotor adaptations and motor coordination strategies under painful conditions, suggesting that pain may reshape motor control at both cortical and muscular levels.

## Declaration of Generative AI and AI-assisted technologies in the writing process

During the preparation of this work the authors used “Le Chat” developed by Mistral AI in order to improve syntax. After using this tool, the authors reviewed and edited the content as needed and took full responsibility for the content of the publication.

**Ethical Publication Statement**.

We confirm that we have read the Journal’s position on issues involved in ethical publication and affirm that this report is consistent with those guidelines. None of the authors has any conflict of interest to disclose.

## CRediT authorship contribution statement

**Arnaud Duport:** Writing – review & editing, Writing – original draft, Visualization, Validation, Supervision, Software, Project administration, Methodology, Investigation, Formal analysis, Data curation, Conceptualization. **Gaspard Diotalevi:** Writing – review & editing, Writing – original draft, Project administration, Methodology, Data curation. **Pierre Morel:** Writing – review & editing, Writing – original draft, Visualization, Methodology, Investigation, Formal analysis, Conceptualization. **Flore Le Blanc:** Writing – review & editing, Writing – original draft. **Guillaume Léonard:** Writing – review & editing, Writing – original draft, Project administration, Methodology. **Hervé Devanne:** Writing – review & editing, Writing – original draft, Project administration, Methodology, Investigation, Data curation, Conceptualization.

## Funding

This work did not receive specific funding.

## Declaration of competing interest

The authors declare that they have no known competing financial interests or personal relationships that could have appeared to influence the work reported in this paper.

## Data Availability

Data will be made available on request.
